# Fitness consequences of outgroup conflict

**DOI:** 10.7554/eLife.74550

**Published:** 2022-07-14

**Authors:** Ines Braga Goncalves, Amy Morris-Drake, Patrick Kennedy, Andrew N Radford

**Affiliations:** 1 School of Biological Sciences, University of Bristol Bristol United Kingdom; Max Planck Institute for Evolutionary Anthropology Germany; University of St Andrews United Kingdom

**Keywords:** contest, group living, intergroup conflict, reproductive success, social evolution, survival

## Abstract

In social species across the animal kingdom, conspecific outsiders threaten the valuable resources of groups and their members. This outgroup conflict is recognised as a powerful selection pressure, but we argue that studies explicitly quantifying the fitness consequences need to be broader in scope: more attention should be paid to delayed, cumulative, and third-party fitness consequences, not just those arising immediately to group members involved in physical contests. In the first part of this review, we begin by documenting how single contests can have survival and reproductive consequences either immediately or with a delay. Then, we step beyond contests to describe fitness consequences that can also result from interactions with cues of rival presence and the general landscape of outgroup threat, and beyond single interactions to describe cumulative effects of territorial pressure and elevated outgroup-induced stress. Using examples from a range of taxa, we discuss which individuals are affected negatively and positively, considering both interaction participants and third-party group members of the same or the next generation. In the second part of the review, we provide suggestions about how to move forward. We highlight the importance of considering how different types of outgroup conflict can generate different selection pressures and of investigating variation in fitness consequences within and between species. We finish by discussing the value of theoretical modelling and long-term studies of natural populations, experimental manipulations, and meta-analyses to develop further our understanding of this crucial aspect of sociality.

## Introduction

**Figure 1.**
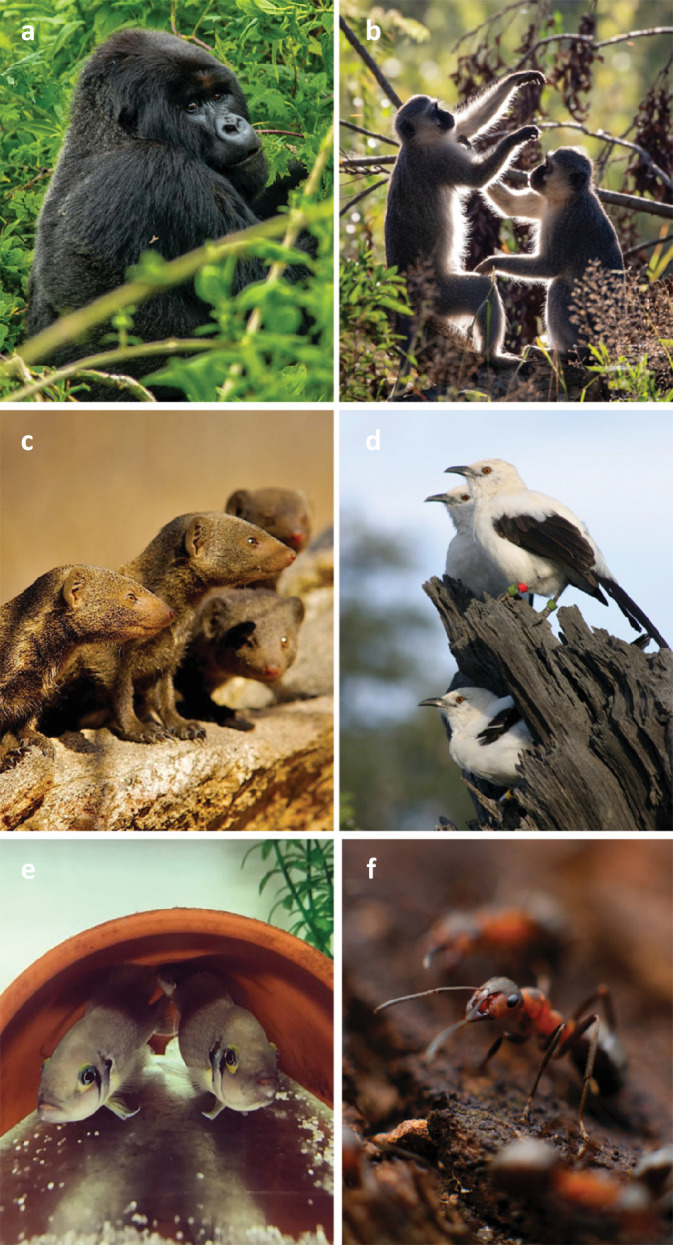
Outgroup conflict occurs in social species throughout the animal kingdom, including (**a**) mountain gorillas (*Gorilla beringei beringei*), (**b**) vervet monkeys (*Chlorocebus pygerythrus*), (**c**) dwarf mongooses (*Helogale parvula*), (**d**) pied babblers (*Turdoides bicolor*), (**e**) daffodil cichlids (*Neolamprologus pulcher*) and (**f**) fire ants (*Solenopsis invicta*). (**d**) Courtesy of Andrew Radford, with permission to publish under a Creative Commons Attribution License. (**e**) Courtesy of Ines Braga Goncalves, with permission to publish under a Creative Commons Attribution License.

Outgroup conflict is widely discussed as a powerful selection pressure in social evolution, but studies quantifying fitness consequences have been somewhat limited in scope. In social species across a broad range of taxa (Figure 1), conspecific outsiders (see Glossary) threaten the valuable resources (e.g. territories, food, mates, and breeding positions) of groups and their members (Christensen and Radford, 2018; Beehner and Kitchen, 2007; Radford et al., 2016). Threats may come from single outsiders, same-sex coalitions, or rival groups; when groups compete, all or just a subset of individuals may participate. We use ‘outgroup’ conflict to encompass all scenarios involving a threat from conspecific outsiders, with ‘intergroup’ conflict the subset of those involving conflict between rival groups. Theoretical work indicates the selective importance of outgroup conflict in the evolution of within-group dynamics, cooperation, territoriality, and social structure (Alexander and Bargia, 1978; Choi and Bowles, 2007; Gaston, 1978; Rusch, 2014). By contrast, the traditional focus of empirical research has been on behaviour during contests between rivals (e.g. how different group members contribute, the costs and benefits of participation, what factors affect the level of escalation and who wins; Arseneau-Robar et al., 2016; Batchelor and Briffa, 2011; Beehner and Kitchen, 2007; Radford, 2003) and, more recently, broader behavioural changes arising as an immediate consequence of outgroup interactions (e.g. changes in individual vigilance and foraging decisions, within-group affiliation, and group movement patterns; Birch et al., 2019; Braga Goncalves and Radford, 2019; Crofoot, 2013; Morris-Drake et al., 2019; Preston et al., 2021; Radford, 2008). Fitness assessments are crucial in general to elucidate selection pressures; in the case of outgroup conflict, they would also help to bridge the gap between theoretical work considering lasting evolutionary differences between species and empirical work considering plastic and ephemeral behavioural responses within species. However, studies that explicitly quantify fitness consequences in the context of outgroup conflict have, to date, focused mostly on those arising immediately from physical contests (for exceptions, see Kerhoas et al., 2014; Lemoine et al., 2020; Rudolph and McEntee, 2016; Thompson et al., 2017).

Beyond the immediate negative impacts of physical contests, broader attention needs to be paid to delayed, cumulative, and third-party fitness consequences, both negative and positive, that arise from living in a landscape of outgroup threat. Adversarial interactions with outsiders, especially if they escalate to physical violence, can lead to immediate direct costs to survival or reproductive success (e.g. loss of life or breeding position; Batchelor and Briffa, 2011; Thompson et al., 2017; Wrangham et al., 2006). However, we argue that consideration of fitness consequences should expand more systematically in three non-exclusive ways. First, as with predation (Creel and Christianson, 2008), the influence of outsiders is likely not restricted to confrontations between animals. For instance, there can be behavioural and hormonal effects of encountering secondary cues (e.g. faecal deposits) of rival presence (Christensen et al., 2016; Morris-Drake et al., 2019) and as a result of the overall risk of outgroup conflict (e.g. the number of territorial neighbours or the likelihood of intrusions; Lemoine et al., 2020; Radford, 2010; Samuni et al., 2020; Schoof and Jack, 2013), which could translate into fitness consequences. Second, in addition to the immediate effects of individual contests, there could be knock-on consequences from contest-related occurrences such as injuries and takeovers (Packer and Pusey, 1983; Schneider-Crease et al., 2020), from changes in behaviour or space use (Crofoot, 2013; Mares et al., 2012) and from the cumulative effects of multiple events (Isbell et al., 1990; Mosser and Packer, 2009). These could affect the fitness of both those directly involved and third-party individuals in the current and subsequent generations (Brunton, 2013; Goldstein et al., 1998; Noguera et al., 2017). Third, a particular event or scenario can have different consequences (including some that are positive) for different individuals within a group. For example, the takeover of a breeding position is most costly to the usurped individual but is positive for the incomer and may affect third-party opposite-sex (e.g. through improved reproductive opportunities) and same-sex (e.g. due to eviction) group members (Balshine et al., 1998; Clutton-Brock et al., 2001).

Here, we describe many of the myriad ways that outgroup conflict could have fitness consequences and what we believe is needed to increase our understanding of this relatively neglected aspect of sociality. There is increasing recognition that not all interactions with conspecific outsiders necessarily entail conflict (Furuichi, 2020; Pisor and Surbeck, 2019): in some species, such as bonobos (*Pan paniscus*), many intergroup encounters are described as peaceful (Fruth and Hohmann, 2018; but see Cheng et al., 2021); whilst in other species, some intergroup interactions at least are characterised by tolerance and/or are about just information exchange (Hashimoto et al., 2020; Radford and du Plessis, 2004). However, we know far less about these types of encounters and their consequences (Van Belle et al., 2020), and there may be elements of conflict even in seemingly tolerant or peaceful exchanges. Thus, our primary focus is a conflict perspective.

Our review has two main parts. In the first part, we describe the full range of potential fitness consequences that could arise from outgroup conflict. We begin by documenting the potential survival and reproductive consequences of single contests, which is the most-commonly considered scenario. Then, we step beyond contests to describe fitness consequences that can also result from interactions with cues of rival presence and the general landscape of outgroup threat, and beyond single interactions to describe cumulative effects of territorial pressure and elevated outgroup-induced stress. We discuss which individuals are affected negatively and positively, using illustrative examples from a range of taxa; we do not present a comprehensive review of the literature. To complement the text presentation of the core ideas, we split the concepts into examples of fitness consequences that arise directly to individuals (immediately, with a delay and cumulatively; Table 1) and those that arise to third-party group members (of the same or subsequent generations; Table 2). In the second part of the review, we provide suggestions about how to move the research field forward. First, we highlight the importance of considering how different types of outgroup conflict can generate different selection pressures and of investigating variation in fitness consequences within and between species. Then, we discuss the value of both theoretical modelling and empirical work, including long-term studies of natural populations, experimental manipulations, and interspecific meta-analyses.

**Table 1.** Potential ways in which outgroup conflict may have immediate, delayed, and cumulative consequences for the survival and reproductive success (RS) of individuals directly affected. Examples are those of outgroup effects; where demonstrated, they also include the ensuing fitness consequences but in some cases, those have yet to be quantified.

Outgroup effects	Potential fitness consequences	Examples
(a) Immediate consequences		
Death of adult	Decreased survival	During intercolony interactions in dampwood termites (*Zootermopsis nevadensis*), founding reproductives are targeted and killed (Thorne et al., 2003).
Death of offspring	Decreased survival	In fights between rival groups of banded mongooses (*Mungos mungo*), pups are the most common victims (Nichols et al., 2015).
Extra-group mating	Increased RS of external male; decreased RS of cuckolded male; increased RS (better genes, unrelated partner) for female	Subordinate female common marmosets (*Callithrix jacchus*) sneak matings with outgroup males whilst other group members are engaged in intergroup contests (Lazaro-Perea, 2001).
Female transfer	Decreased RS for male(s) in original group; increased RS for male(s) in new group	Female hamadryas baboons (*Papio hamadryas*) may be kidnapped by rival males during intergroup contests; males from the original group may attempt to recover the females, putting themselves at risk of serious injury (Pines and Swedell, 2011).
Breeder replacement	Increased RS for incoming breeder; decreased RS for usurped breeder	In Arabian babblers (*Turdoides squamiceps*), outsiders frequently take over the breeding position in a group; coalitions of same-sexed individuals are more successful at takeovers than lone individuals (Ridley, 2011).
(b) Delayed consequences		
Injury	Decreased survival and RS	In mountain gorillas (*Gorilla beringei beringei*), attacks on intruding adult males can result in severe injury (Rosenbaum et al., 2016).
Disease / parasite transmission	Decreased survival and RS	Honeybees (*Apis mellifera*) from healthy colonies that rob honey from neighbouring colonies collapsing from Varroa mite infestations inadvertently carry the mites back to their own colonies (Peck and Seeley, 2019).
Avoidance of area	Decreased survival and RS	Baboon (*Papio cynocephalus*) groups that lose intergroup contests avoid the area around the encounter location in the following three months (Markham et al., 2012).
Change in behaviour (e.g. movement)	Decreased survival and RS	White-faced capuchin (*Cebus capucinus*) groups that lose intergroup contests move further, faster, and for longer compared to groups that won (Crofoot, 2013).
(c) Cumulative consequences		
Change in territory size	Increased survival and RS for winners; decreased survival and RS for losers	Artificially reducing the colony size of a territorial ant, *Azteca trigona*, resulted in loss of territory (by up to 35%) to neighbours (Adams, 1990).
Stress	Decreased survival and RS	Cortisol levels are higher in chimpanzees (*Pan troglodytes*) on days when the group experiences an intergroup encounter (Samuni et al., 2019); female reproductive success is reduced (increase in inter-birth intervals) when pressure from neighbouring groups, and likely stress, is high (Lemoine et al., 2020).

**Table 2.** Potential ways in which outgroup conflict may have consequences for the survival and reproductive success (RS) of third-party individuals following an initial effect on others. Examples are those of third-party effects from outgroup conflicts; where demonstrated, they also include the ensuing fitness consequences but in some cases, those have yet to be quantified.

Outgroup effect	Third-party effect	Potential fitness consequences	Examples
(a) Same generation			
Change in breeder	Access to unrelated potential mate	Increased breeding opportunities for opposite-sex group members	Subordinate female meerkats (*Suricata suricatta*) are more likely to reproduce when there are unrelated males in the group (Clutton-Brock et al., 2001).
	Changes to female reproductive output	Reduced fertility	Following male takeovers, female African lions (*Panthera leo*) that lose dependent young to infanticide take about 3.5 months longer to conceive again relative to females that lose young under other circumstances (Packer and Pusey, 1983).
	Infanticide	Decreased RS for parents; increased RS for incoming male	Male takeovers in geladas (*Theropithecus gelada*) are associated with a 32-fold increase in rates of infant death and a halving of inter-birth intervals in females that lost their infants following the takeover (Beehner and Bergman, 2008).
	Eviction of adults	Decreased survival and RS for evicted individuals	Following takeovers in Arabian babblers (*Turdoides squamiceps*), same-sex subordinates are often evicted from the group (Ridley, 2011).
Change in group size	More groupmates	Decreased risk of group extinction	In several ant species, including the honey ant *Myrmecocystus mimicus* and the fire ant *Solenopsis invicta*, workers in starting colonies raid nearby conspecific nests for brood (intraspecific slave-making), with colonies that have the most workers being most likely to prevail (Pollock and Rissing, 1989).
	Fewer groupmates	Decreased survival and RS	Death of a groupmate during an outgroup contest reduced the resource-holding potential of a spotted hyaena (*Crocuta crocuta*) group, resulting in substantial loss of territory to competing groups and individuals being more vulnerable to heterospecific competitors and predators (Henschel and Skinner, 1991).
(b) Next generation			
Time and energy in contests	Reduced quality of parental care	Decreased offspring survival	Pied babbler (*Turdoides bicolor*) groups, especially those with fewer members, leave nests exposed to predators and nestlings to go hungry during territory defence against neighbouring groups, resulting in lower reproductive success (Ridley, 2016).
Change in breeder	Infanticide	Decreased offspring survival	In crested macaques (*Macaca nigra*), group takeovers by immigrant males are associated with a near tripling in the probability of infant mortality (Kerhoas et al., 2014).
	Eviction of independent young	Decreased survival for evicted individuals	Following a pride takeover, incoming male African lions often evict independent sub-adults; young males rarely disperse successfully, invariably resulting in premature deaths (Elliot et al., 2014).
Parental stress	Decreased offspring quality	Decreased infant survival	In chimpanzees (*Pan troglodytes*), the level of neighbour pressure experienced during pregnancy is negatively associated with subsequent infant survival (Lemoine et al., 2020).
	Reduced offspring size	Reduced future RS	Daffodil cichlid (*Neolamprologus pulcher*) groups experiencing chronic outgroup conflict produce young with lower survivorship and smaller body size (Braga Goncalves and Radford, 2022); surviving young likely incur fitness costs because adult body size is a key determinant of dominance and fecundity in this species (Wong and Balshine, 2011).

### Range of fitness consequences

Our aim in this section is to describe and discuss the variety of fitness consequences that can result from outgroup conflict. Some of these (e.g. targeted killing of rivals by raiding parties) may be unique to this type of social interaction, whilst others (e.g. infanticide) can arise in several contexts. Some (e.g. loss of a breeding position) predominately occur due to threats from individual outsiders or same-sex coalitions, whilst others (e.g. loss of territory space) are because of conflict with rival groups. But we believe that a description of the wide range of potential fitness consequences is an important starting point for understanding outgroup conflict as a general selection pressure. This is because a narrow focus only on contests or just one type of fitness consequence risks missing competing or balancing pressures—selection does not just act on traits that help to outcompete outsiders but also on many others relating to, for instance, the minimisation of risk, moderation of stress, and dispersal decision-making—and because threats from different types of conspecific outsiders (individuals, coalitions, whole groups) can occur in the same species at different times and can affect group members differently.

### Consequences of single contests

**Figure 2.**
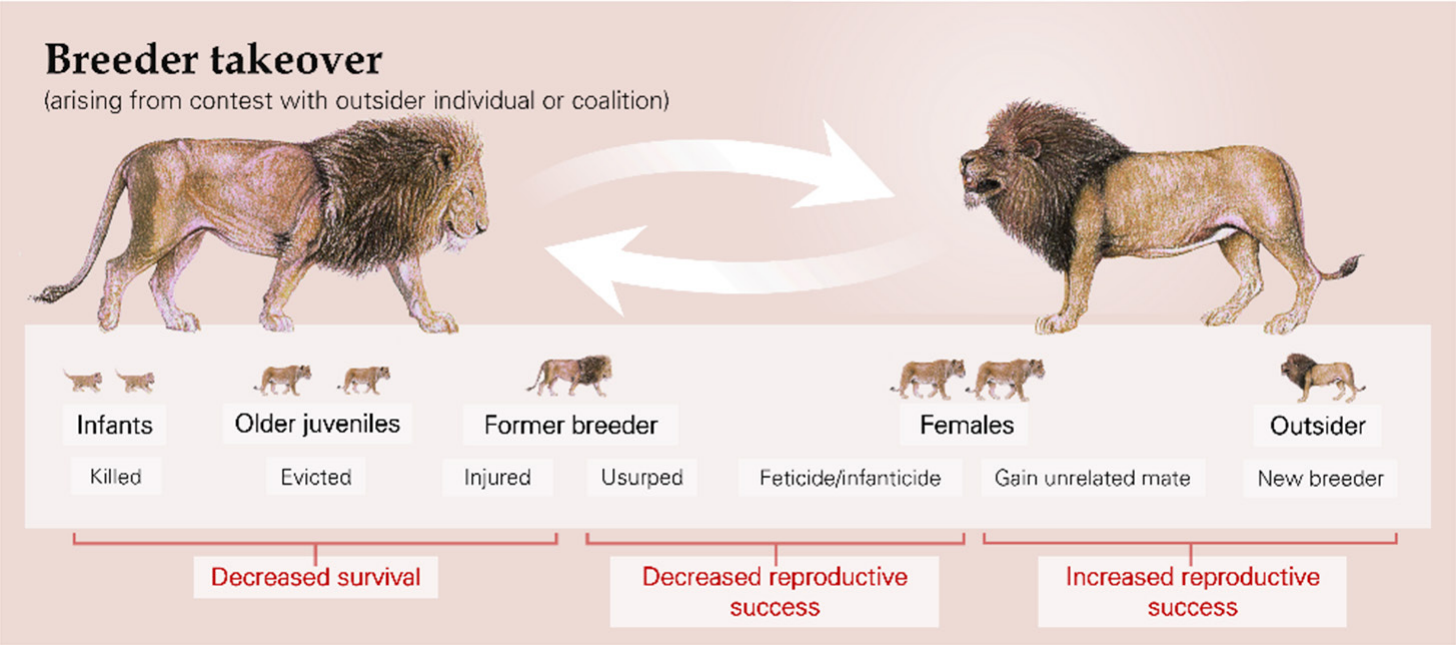
The enforced takeover of a breeding position by one or more outsiders can have a series of immediate and delayed fitness consequences, for both contest participants and for same- and next-generation third-party individuals, as illustrated by African lions (*Panthera leo*). Lion artwork is by Martin Aveling and is not available under the terms of a Creative Commons Attribution licence; further reproduction of these images requires permission from the copyright holder.

Outgroup contests can have immediate fitness consequences for those involved (Table 1a): there can be loss of life, extra-group mating, transfer of females, and replacement of breeders. The most extreme example is targeted killing: in chimpanzees (*Pan troglodytes*), for instance, coalitions of group members undertake coordinated incursions into neighbouring territories seemingly with the intention of attacking rivals (Goodall et al., 1979; Wilson et al., 2014), whilst raids to kill offspring in rival groups occur in species such as banded mongooses (*Mungos mungo*) and greater anis (*Crotophaga major*) (Cant et al., 2016; Strong et al., 2018). More commonly, the death of participating adults or juveniles accidentally caught in the melee arises as a by-product of escalated physical contests (Dyble et al., 2019; Thorne et al., 2003; Zahavi, 1990). There is also the possibility that engagement in fighting behaviour incurs an increased predation risk as participants are distracted (Hess et al., 2016; Jakobsson et al., 1995). Contest-related extra-group mating, which have negative reproductive consequences for the cuckolded male but are positive for the outsider and for the female if she gains better genes or access to an unrelated partner, can arise in two main ways. In some species, such as alpine marmots (*Marmota marmota*) and meerkats (*Suricata suricatta*), males ‘rove’ between groups specifically seeking mating opportunities (Lardy et al., 2015; Young et al., 2007); in other species, such as banded mongooses and common marmosets (*Callithrix jacchus*), individuals from different groups sneak matings whilst others are occupied in outgroup contests (Johnstone et al., 2020; Lazaro-Perea, 2001). Longer-lasting reproductive consequences result from the transfer of females between groups, as seen in various primate species (Breuer et al., 2016; Pines and Swedell, 2011), and enforced takeovers of breeding positions by outsiders (Figure 2), which occur across many taxa (primates: Beehner and Bergman, 2008; carnivores: Packer and Pusey, 1983; ungulates: Rubenstein and Nuñez, 2009; rodents: Hackländer and Arnold, 1999; birds: Ridley, 2011). A takeover follows a particular contest event, though that can sometimes be the culmination of a series of skirmishes over an extended period (Dunbar, 1987; Sicotte et al., 2017), with the usurped individuals losing future (and potentially current; see below) reproductive success unless they themselves can take over another group in turn.

Outgroup contests can also generate a variety of knock-on fitness consequences. First, there can be delayed consequences for contest participants specifically (Table 1b). For instance, physical confrontations with outsiders can lead to injuries in a wide range of species (e.g. primates: Aureli et al., 2006; Rosenbaum et al., 2016; carnivores: Jordan et al., 2017; Mosser and Packer, 2009; mongooses: Dyble et al., 2019; Thompson et al., 2017; birds: Hannon et al., 1985; insects: Batchelor and Briffa, 2011; Rudolph and McEntee, 2016). Injured animals likely have a greater mortality rate and reduced reproductive performance (Bernardo and Agosta, 2005; Krause et al., 2017; Wilson, 1992). The second category of knock-on fitness consequences are those resulting in third-party individuals; that is, adult group members (Table 2a) and offspring (Table 2b) who were not necessarily directly involved in the original outgroup contest. For instance, in primate species where females may be kidnapped or voluntarily move to a rival group during contests, their own-group males may aggressively herd or coerce them to remain (Breuer et al., 2016). Another possibility is that lethal fights (see above) create a breeding vacancy that a subordinate group member benefits from filling (Johns et al., 2009). In at least one case, current non-breeders increase the likelihood of this occurrence: dry wood termite (*Cryptotermes secundus*) workers are believed to tunnel through to the next colony to incite an intergroup conflict, increasing the prospect of their king and queen being killed and a breeding vacancy arising (Korb and Roux, 2012).

Other striking examples of third-party consequences follow a change in breeder (Figure 2). This event may be beneficial to members of the opposite sex if there is now an unrelated dominant individual with whom they can mate. For example, in geladas (*Theropithecus gelada*), subordinate females are more likely to mature sexually in the two months following a male breeder takeover than in the preceding two months (Beehner and Lu, 2013), although accelerated sexual maturity may not always be beneficial. There are also potential negative consequences for existing group members. For instance, the remaining breeder may suffer a decrease in reproductive output; newly established pairings may produce fewer offspring compared to those who have been together a long time, as seen in alpine marmots, Azara’s owl monkeys (*Aotus azarai*), and pied babblers (*Turdoides bicolor*) (Fernandez-Duque and Huck, 2013; Lardy et al., 2015; Wiley and Ridley, 2018). The loss of a valuable ally could, in principle, result in a lower position in the dominance hierarchy and thus fewer mating opportunities or lowered survival (Cheney and Seyfarth, 1987). In a range of species, including African lions (*Panthera leo*), ursine colobus (*Colobus vellerosus*), and geladas, incoming male breeders kill dependent offspring sired by their predecessors to bring females into oestrus sooner (Packer and Pusey, 1983; Schneider-Crease et al., 2020; Sicotte et al., 2017); females may also exhibit higher abortion rates (Roberts et al., 2012; Zipple et al., 2017) and reduced fertility (Packer and Pusey, 1983) following male takeovers. Infanticide and feticide are costly for the reproductive success of the parents but benefit the new breeding males. Incoming breeders of both sexes sometimes evict existing group members: former breeders and other adults may be evicted as potential rivals, as seen in the cichlid *Neolamprologus pulcher* and Florida scrub-jays (*Aphelocoma coerulescens*) (Balshine et al., 1998; Goldstein et al., 1998), whilst independent offspring may be driven to disperse early (Elliot et al., 2014). In principle, some existing group members may also choose to disperse if the presence of a new breeder reduces inclusive fitness benefits (Eikenaar et al., 2007; Rowley and Russell, 1990; Spong et al., 2008). Individuals that leave or are evicted can suffer fitness consequences because being alone or in a small splinter group likely results in increased predation, reduced foraging success, and fatal encounters with rival groups (Cant et al., 2001; Kingma et al., 2016; Ridley et al., 2008); there is clear evidence that spending time alone has a negative effect on longevity in lions and meerkats (Cram et al., 2018; Elliot et al., 2014).

Outgroup contests can also have knock-on fitness consequences for all group members, rather than just specific individuals, because of group-size changes (Table 2). Contest-related deaths of adults and offspring, infanticide by incoming breeding males, female transfers, and dispersal or eviction following a change in breeder (details above) can all lead to a reduction in group size. Conversely, group-size increases can arise when a breeding individual is usurped by a same-sex coalition (Bygott et al., 1979; Ridley, 2011), when there is kidnapping of young from rival groups as seen in white-winged choughs (*Corcorax melanorhamphos*), banded mongooses and pied babblers (Heinsohn, 1991; Müller and Bell, 2009; Ridley et al., 2022), and when intraspecific slave-raiding occurs in insects such as the honey ant *Myrmecocystus mimicus* and the fire ant *Solenopsis invicta* (Bartz and Hlldobler, 1982; Tschinkel and Howard, 1983). Changes in group size can have a variety of fitness consequences, with the positive effects of increased group size the reverse of the negative ones arising from a reduced group size that we describe here. Individuals in smaller groups may suffer a general increase in mortality risk both from predation and starvation, although smaller groups might be less easily detected by predators and may have less competition among group members for limited resources (Krause and Ruxton, 2002). Fewer group members can also mean a reduced fighting strength when competing with conspecific rivals; relative group size is known to play an important role in intergroup contest outcomes, as evidenced from meerkats and green woodhoopoes (*Phoeniculus purpureus*) (Dyble et al., 2019; Radford and du Plessis, 2004). Moreover, loss of offspring can reduce the motivation to fight (Dyble et al., 2019). In cooperatively breeding species, the loss of helpers likely has negative effects on the inclusive fitness of breeders and other group members because helper number is often positively related to reproductive success (Brown et al., 1982; Russell et al., 2007; Taborsky et al., 2007). The loss of adults or offspring is especially costly for small groups that are more sensitive to a reduction in group size; in cooperatively breeding species, the maintenance of a critical size is vital to avoid group extinction (Courchamp et al., 1999; Taborsky et al., 2005).

### Beyond contests

**Figure 3.**
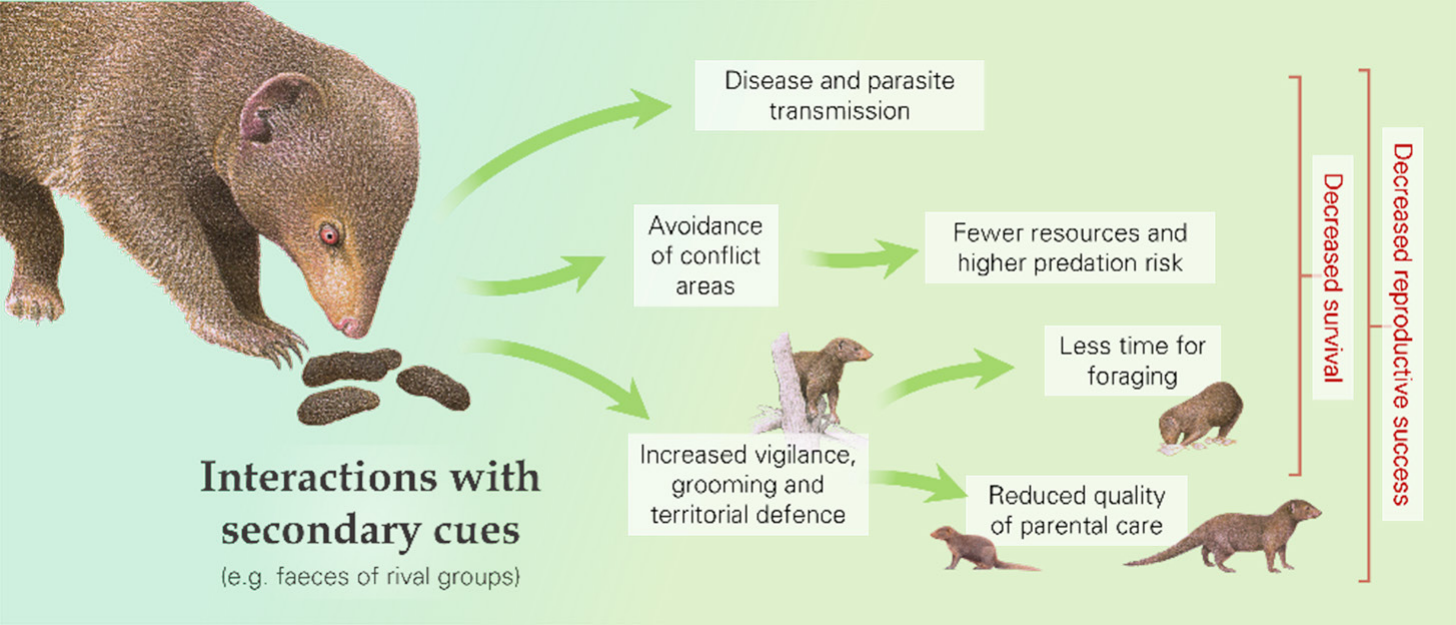
Interactions with secondary cues of rival groups (as well as with the outsiders themselves) can cause behavioural changes and increase the risk of disease and parasite transmission, with downstream fitness consequences, as illustrated by dwarf mongooses (*Helogale parvula*). Mongooses artwork is by Martin Aveling and is not available under the terms of a Creative Commons Attribution licence; further reproduction of these images requires permission from the copyright holder.

Some fitness consequences of outgroup conflict may arise not only from engagement in contests but also from interactions with secondary cues of rivals (Figure 3; Table 1b). Close encounters with outsiders and inspection of rival faeces, urine, and other secretions, for instance, could lead to disease and parasite transmission (Brown and Brown, 2004; Craft et al., 2011; Drewe, 2010; Nolan and Delaplane, 2016). As a specific example, modelling of disease transmission in African lions demonstrates the importance of contact between neighbouring prides (Craft et al., 2011). Susceptibility may be further increased in the context of outgroup conflict because social stress is a key factor in the pathogenesis of disease (Padgett et al., 1998; Quan et al., 2001). There are clear survival costs for animals who have contracted a disease or carry a high parasite load: these individuals may be more prone to, for example, lethal infections, starvation, and predation (Milinski, 1985; Robar et al., 2010). Individuals compromised in these ways may also have a lowered reproductive output (Fitze et al., 2004; Scott, 1988).

Interactions with rivals and cues of their presence (e.g. olfactory or auditory indicators) can result in a range of behavioural responses that could have fitness consequences (Figure 3; Table 1b). For instance, there can be alterations in space use: groups might avoid areas where contests typically occur (Markham et al., 2012; Mech and Harper, 2002; Seiler et al., 2017) or might spend more time in border zones to guard against potential intrusions, as seen in green woodhoopoes that are more likely to roost where an intergroup conflict occurred earlier in the day (Radford and Fawcett, 2014). In both cases, this might mean more time spent in areas with fewer food resources, more predators and/or less-preferred sleeping sites, and thus an increased risk of starvation or predation (Crofoot, 2013). In addition to changes in space use, outgroup interactions can cause other behavioural responses. For instance, defensive actions can include greater patrolling and scent-marking (Amsler, 2010; Christensen et al., 2016; Jordan et al., 2007), whilst there may be more general increases in vigilance and intragroup affiliation (Morris-Drake et al., 2019; Radford, 2008; Lemoine et al., 2020; Walker et al., 2016), altered speed of movement, and reduced time spent resting (Christensen et al., 2016; Crofoot, 2013; Mirville et al., 2020). In dwarf mongooses (*Helogale parvula*), for example, presentation of rival-group faeces compared to control faeces resulted in more scent-marking, vigilance, and grooming (Christensen et al., 2016; Morris-Drake et al., 2019). Losing groups of white-faced capuchins (*Cebus capucinus*) moved further and faster, stopped less frequently and were active until later in the evening than groups that won intergroup encounters (Crofoot, 2013). These behavioural changes have likely knock-on consequences in terms of greater energy expenditure and reduced time for foraging, and thus lower body mass, and lessened parental care (Crofoot, 2013; Mares et al., 2012; Morris-Drake et al., 2021), which could influence the reproductive success and survival chances of both adults and dependent young (Table 2).

Many of the studies considering behavioural effects of outgroup conflict have focused on the period immediately following a single interaction (up to 1 or 2 hr in the aftermath), when responses are most likely due to elevated stress (Culbert et al., 2021; Samuni et al., 2020; Schoof and Jack, 2013) or exclusion from territorial areas (Crofoot, 2013; Mirville et al., 2020). Fitness consequences from such short-term, single occurrences might be relatively small, in at least some instances. However, there is also some evidence for longer-lasting behavioural effects of single events (Dyble et al., 2019; Radford and Fawcett, 2014) and that the overall threat of outgroup conflict (a ‘landscape of fear’) may cause avoidance of likely conflict areas (Markham et al., 2012; Seiler et al., 2017) and behavioural changes when in such locations (Radford, 2010). Moreover, there could be a cumulative build-up from multiple outgroup events which results in behavioural changes not just in the immediate aftermath of each interaction, but also more generally to baseline activity (Morris-Drake et al., 2021; Thompson et al., 2020). As these scenarios all increase the frequency and magnitude of behavioural changes, they enhance the likelihood of fitness consequences arising from them.

### Beyond single interactions

In addition to cumulative behavioural effects (see above), a build-up of outsider pressure over time can lead to changes in territory ownership or size. In extreme cases, a group might be usurped from its whole territory either by neighbours or groups from further afield (Isbell et al., 1990; Ligon and Ligon, 1990; Mitani et al., 2010). For instance, Goodall, 1986 famously documented how the Kasekela community of chimpanzees at Gombe National Park took over the territory of the neighbouring Kahama community after a series of lethal attacks. More commonly, a group may lose part of its territory to a stronger neighbour, as seen in rattling cisticolas (*Cisticola chiniana*), vervet monkeys (*Chlorocebus pygerythrus*), chowchillas (*Orthonyx spaldingii*), and lions (Carlson, 1986; Isbell et al., 1990; Jansen, 1999; Mosser and Packer, 2009). Losing groups then have access to areas of reduced quality and/or less familiarity, and thus individuals have potentially lessened survival chances and reproductive success (Table 1c). Survival might be reduced due to reliance on poorer quality food resources, more time where there is a higher predation risk (in terms of predator numbers and less familiarity with escape options) or limited availability of safe sleeping sites (Crofoot, 2013; Isbell et al., 1990; Markham et al., 2012; Mosser and Packer, 2009). Reduced access to valuable resources could also have negative impacts on both current and future reproductive success, as documented for mud crabs (*Panopeus herbstii*), Seychelles warblers (*Acrocephalus sechellensis*), and chimpanzees (Griffen and Norelli, 2015; Komdeur and Edelaar, 2001; Thompson et al., 2007). These consequences are qualitatively similar to at least some arising from temporary avoidance of areas (see ‘Beyond contests,’ above). However, when there is a permanent change in territory ownership, there are also benefits to those groups gaining additional resources, who likely experience positive effects on survival and reproductive success. One further benefit of increasing territory size in some bird species, such as Seychelles warblers and Florida scrub jays, is that sons can ‘bud’ off part of the territory and so begin reproducing independently (Komdeur and Edelaar, 2001; Stallcup and Woolfenden, 1978).

**Figure 4.**
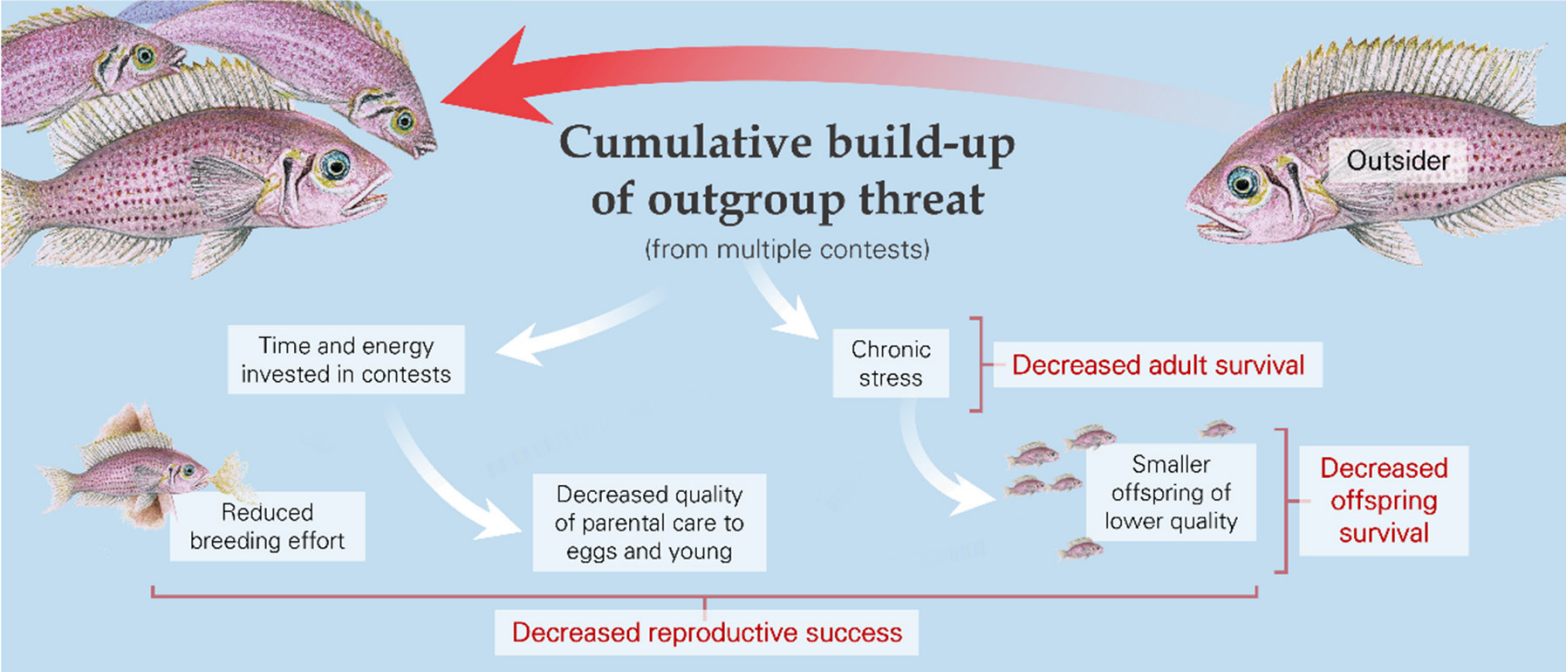
The cumulative pressure from outsiders, whether from multiple contests or the general threat of conflict, can affect adult reproduction and offspring number and characteristics, as illustrated by the daffodil cichlid (*Neolamprologus pulcher*). Fish artwork is by Martin Aveling and is not available under the terms of a Creative Commons Attribution licence; further reproduction of these images requires permission from the copyright holder.

The cumulative build-up of outgroup threat likely generates chronic stress (Samuni et al., 2019), with survival and reproductive consequences for the affected individuals (Figure 4; Table 1c). Chronic stress is associated with reduced body condition and increased chances of mortality (Campos et al., 2021; Pride, 2005; Wey et al., 2014) due to, for example, increased susceptibility to predation (Romero et al., 2009; Vuarin et al., 2019). Chronic stress may also reduce reproductive investment and success: it can have negative effects on courtship activity (Romero-Diaz et al., 2019; Schreck, 2010), breeding rates (Dulude‐de Broin et al., 2020; Mileva et al., 2011), fecundity (O’Brien et al., 2018; Schreck, 2010), egg size and composition (Ensminger et al., 2018; Henriksen et al., 2013), and hatching and fledging success (Cyr and Michael Romero, 2007; Eriksen et al., 2015; Kleist et al., 2018). Many of these effects can occur in the same species (Zanette et al., 2011). There has been only a limited amount of research examining the reproductive consequences of outgroup conflict directly. A recent observational study on chimpanzees found that an increased cumulative pressure from intergroup conflict is correlated with longer inter-birth intervals and reduced offspring survival (Lemoine et al., 2020). Experimental work with cichlid fish has demonstrated that an increase in outgroup threat can drive longer inter-clutch intervals, cause females to produce relatively smaller eggs with less protein, and result in fewer offspring surviving to one month post-hatching (Braga Goncalves & Radford, In revision). The consequences are not necessarily always negative, however, as outgroup threat has been found to be correlated with reduced foetal mortality in crested macaques (*Macaca nigra*) and banded mongooses (Kerhoas et al., 2014; Thompson et al., 2017) and with increased pup survival in dwarf mongooses (Morris-Drake, 2021).

As with individual contests (see ‘Consequences of single contests,’ above), there can be knock-on consequences of cumulative outgroup effects for third-party individuals – most notably, in this case, for offspring (Figure 4; Table 2b). These could arise through maternal effects, due to conflict-induced stress in mothers (Brunton, 2013; Culbert et al., 2021). In general, offspring from smaller eggs and those with, for example, higher levels of corticosterone might be smaller, be of lower quality, and have learning difficulties (Dantzer et al., 2019; McCormick, 1998; Roche et al., 2012). Stress, as well as engagement in conflict-related activities, could also decrease the quality of offspring care by parents and, in the case of cooperative breeders, non-breeding helpers (Mares et al., 2012; Stein and Bell, 2012). Carers might abandon nests, whilst offspring could be at greater risk of starvation if there is reduced provisioning and at greater risk of predation as a direct consequence of decreased protection or indirectly if smaller offspring are more vulnerable (Ahnesjo, 1992; Ridley, 2016; Vitousek et al., 2014; Vitousek et al., 2018). Moreover, early-life stress on offspring and lower growth has lasting effects: there is increasing evidence that developmental trajectories shape the physiology and behaviour of adults, with major effects on survival and reproductive success (Cram et al., 2017; English et al., 2013; Marshall et al., 2017; Noguera et al., 2017; Royle et al., 2005).

### Moving forward

In the first part of this review, we have deliberately described the full range of fitness consequences that could potentially arise from outgroup conflict, to avoid too narrow a perspective. But moving forward, identification of selection pressures will require careful investigation of the variation that is likely apparent at different levels – depending on both the type of threat (e.g. that presented by an individual outsider, a same-sex coalition, or a whole group) and that within and between species in, for instance, the frequency and intensity of conflict. We argue that to develop this understanding will require complementary and varied theoretical and empirical approaches.

### Variation in fitness consequences

Ultimately, fitness consequences lead to selection pressures, and understanding those relating to outgroup conflict requires consideration of the type of outsider with which a group is engaged. Most obviously, conflict with whole groups likely has fitness consequences for all group members to a greater or lesser extent; if for instance, part of a territory is usurped by others (Carlson, 1986; Isbell et al., 1990; Mosser and Packer, 2009), then there will be fewer resources for all. By contrast, conflict with a single outsider seeking breeding opportunities (Balshine et al., 1998; Lardy et al., 2015; Young et al., 2007) likely carries particularly high costs for one or a subset of group members; indeed, there may be fitness benefits to others. Moreover, whilst intergroup conflict typically involves repeated interactions between the same groups of conspecifics, often in specific (contested) areas (Radford, 2010), competition with single outsiders is more likely to be with different individuals and the contest location probably varies to a greater extent. Consequently, the evolved mechanisms that allow individuals to regulate the costs and benefits of these conflicts and the resulting fitness consequences likely differ in important ways, which in turn affects selection pressure on, for instance, cognitive abilities (Ashton et al., 2020). Identifying the different pressures on individuals is non-trivial, not least because groups in many species face threats from both individuals and other groups, and indeed possibilities on the continuum in between (e.g. coalitions of same-sex rivals) (Dyble et al., 2019; Ridley, 2016; Young et al., 2007), but is important if the evolutionary consequences of outgroup conflict are to be determined. Furthermore, as our understanding develops about the breadth of outgroup interactions seen—i.e., that they may be tolerant and affiliative as well as antagonistic (Furuichi, 2020; Pisor and Surbeck, 2019; Van Belle et al., 2020)—so should our investigation of how that influences the wide range of potential fitness consequences and resulting selection pressures.

Beyond the broad nature of the outsider threat, we believe that a focus of future work should be investigations of the ecological and social reasons for variation in fitness consequences between different species, groups, and individuals (for a full review, see Morris-Drake et al., 2022). Considerable interspecific variation exists in the frequency and intensity of outgroup encounters, which likely influences their relative importance in driving individual fitness. For example, in species such as green woodhoopoes, most intergroup contests are resolved through vocal signalling and thus do not result in deaths (Radford, 2003); in striking contrast, interactions between rival banded mongoose groups usually escalate into physical combat, with 10% of adult and 20% of juvenile deaths attributed to this cause (Johnstone et al., 2020). Variation in contest frequency and intensity, as well as differences in social structure and dispersal, likely also affect rates of extra-group mating and breeder replacements, the prevalence of disease and parasite transmission, within-group behaviour and relationships, and chronic stress levels. At a group level, there can be established dominance hierarchies with clear fitness benefits to members of more dominant groups. In lions, for example, dominant groups have higher-quality territories, which are associated with greater female reproductive success (Mosser and Packer, 2009). However, dominant groups do not always win against more subordinate rivals—interaction location, role (attacker or defender), and motivation can all have an influence (Crofoot et al., 2008; Furrer et al., 2011; Strong et al., 2018). Moreover, winners may suffer important costs, as evidenced in acacia ants (*Crematogaster mimosae*): in outlasting or killing rivals, victors expend valuable time and energy and may lose resource-holding capacity (i.e. suffer their own losses in numbers), meaning a reduced ability to defend themselves from predators, parasites or subsequent attacks from conspecifics (Rudolph and McEntee, 2016). There is also likely great variation between group members in the fitness consequences arising from the same outgroup contest, often due to differences in contest participation. For instance, aggressive intergroup interactions in banded mongooses may be initiated by females moving into the territory of a neighbouring group; the females may gain extra-group matings whilst their own-group males are distracted in battle and suffer the physical costs of the contest (Johnstone et al., 2020). In terms of chronic effects, sex differences in regulation of the vertebrate hypothalamic-pituitary-adrenal/interrenal axes (Young et al., 2007) mean that there are likely differences in how males and females respond to the stress of outgroup conflict (Culbert et al., 2021). Determining these different, competing and balancing fitness consequences is key to establishing the selective pressure of outgroup conflict.

### Methods of study

To date, theoretical modelling of outgroup conflict has mainly focused on the determinants of group success in contests (Franks and Partridge, 1993; Johnson and MacKay, 2015), individual variation in participation (Gavrilets and Fortunato, 2014; Schindler and Radford, 2018), and demographic influences on costs and benefits of involvement (Lehmann, 2011; see Rusch and Gavrilets, 2020 for a review). A formal theoretical framework integrating the key factors determining the fitness consequences of outgroup conflict, incorporating cumulative as well as immediate consequences, is yet to be created. Models are also needed to explore how differences in outgroup and ingroup relatedness for different classes of individual, which may be affected by different dispersal patterns and spatial scales of competition, can lead to differences in indirect fitness costs and benefits of outgroup conflict (Micheletti et al., 2020). Translating theory developed in the context of international relations, such as models of border tensions between nations (Konrad and Morath, 2015), to biology may be a productive starting point when considering conflict arising between rival groups (Rusch and Gavrilets, 2020). New theoretical models should aim to make a priori predictions about understudied traits—for example, recent models have developed testable predictions for intragenomic conflict over participation in human intergroup conflict (Micheletti et al., 2017). Ideally, models should be parameterised by existing data, incorporate empirically quantified trade-offs (e.g. vigilance versus foraging; Verdolin, 2006), and allow cost and benefit functions to emerge as a consequence of realistic social and demographic parameters in the model. As with any aspect of behavioural biology, powerful studies combine new theory with empirical tests of the generated predictions (see Johnstone et al., 2020).

Three broad types of empirical study will be useful to test theoretical predictions: long-term observational studies, experimental manipulations, and interspecific comparisons. Because outgroup interactions occur repeatedly, and their effects can be cumulative and transgenerational, long-term datasets from individually identifiable wild animals (Clutton-Brock and Sheldon, 2010) offer a particularly valuable window into fitness consequences (Kerhoas et al., 2014; Lemoine et al., 2020; Thompson et al., 2017). Whilst there is a strong track record of recording behaviour during outgroup interactions, more consistent measurement of fitness consequences for different parties (e.g. winners and losers, different group members) is needed because there can be considerable variation between individuals and situations. Captive-based manipulations allow for precise control over, for example, the presence of outsiders and territorial intrusions, maintenance of similar conditions between groups and across time, detailed tracking of ultimate effects on survival and reproductive success at an individual level, and quantification of transgenerational consequences (including from adaptive and passive maternal effects). However, they are likely only feasible for some taxa such as invertebrates and fish (Batchelor and Briffa, 2011; Braga Goncalves and Radford, 2022). Field manipulations allow testing with maximum ecological realism and in a wider range of taxa. There is potential, for instance, to manipulate the perceived outgroup risk through use of rival cues (Herbinger et al., 2009; Morris-Drake et al., 2019; Preston et al., 2021) or to generate asymmetries in whole-group resource-holding potential, such as by supplementary feeding, temporary removal of group members or territory manipulation (Adams, 1990; Balshine et al., 2001; Kaiser et al., 2015; Powell et al., 2017). Removal of entire groups to manipulate population density and spatial intergroup interaction networks may also be feasible in some cases. But the logistical challenges and ethical considerations (especially as outgroup conflict can cause stress and have lasting consequences, including those that transcend generations; see earlier) mean that manipulations need to be carefully considered and some at least might need to be restricted to invertebrates (Rudolph and McEntee, 2016). Cross-species comparisons will also be valuable as outgroup conflict is a taxonomically widespread and common, yet variable, occurrence. There is scope for synthesising existing datasets from multiple species, identifying common patterns between contexts, and developing a predictive framework to explain interspecific variation in fitness consequences. As a greater understanding of tolerant, as well as competitive, interactions develops, their importance can also be assessed too. Careful categorisation of outsiders and identification of clear metrics and measurable proxies of outgroup conflict that apply across species will be crucial (Ashton et al., 2020).

### Conclusion

Despite outgroup conflict being recognised as a powerful selective pressure, we have argued that more detailed and focused research is needed into its fitness consequences if we are to gain a full understanding of the influence on social evolution. We have focused on organismal societies, with our examples demonstrating the taxonomic breadth of outgroup conflict in the animal kingdom. But this aspect of competition is prevalent across all major transitions (e.g. that from unicells to a multicellular organism), and there is potentially much to gain from greater exchange of ideas between those working on different levels of social organisation (e.g. intra- and intercellular conflict). Moreover, the consequences of outgroup conflict have relevance beyond biology, extending to the fields of anthropology, psychology, economics, and social and political sciences. We, therefore, hope that our review, describing direct and knock-on consequences arising across a range of timeframes and involving positive and negative implications for different individuals, will stimulate future valuable work in a range of disciplines.

### Glossary

Direct consequences: fitness consequences of outgroup contests arising immediately to participants.

Fitness consequences: the effect on survival and reproductive success of an individual; fitness consequences arising from outgroup conflict, interactions, or contests can occur immediately, with a delay or cumulatively, can affect participants or third parties, and can be positive or negative.

Group: a stable aggregation of three or more individuals who tend to remain together and interact with one another more than with other individuals; group members likely have at least some common interests and therefore cooperate to achieve and protect those interests.

Knock-on consequences: consequences of outgroup conflict, interactions, and contests arising with a delay or cumulatively to participants and to third-party group members of the same or the next generation.

Intergroup: a subset of outgroup involving conflict, interactions, or contests between two groups; also referred to in the literature as between-group.

Outgroup conflict: a situation in which the fitness interests of all or some of a group are not aligned and are incompatible with those of one or more conspecific outsiders.

Outgroup contest: a competitive interaction between a group and one or more conspecific outsiders; competition can manifest through signalling displays and/or escalate to physical fights.

Outgroup effects: outcomes of outgroup conflict, interactions, or contests; these may have fitness consequences for participants (Table 1) or third-party group members (Table 2).

Outgroup interaction: the interaction of a group with one or more conspecific outsiders or cues of their presence.

Outgroup threat: the overall threat from outgroup conflict across a landscape.

Outsiders: conspecific individuals that are not part of the focal group; also referred to in the literature as extra-group conspecifics or outgroup individuals.

Participants: group members involved in an outgroup interaction or contest and who may be affected directly.

Third-parties: group members not directly involved in an outgroup interaction or contest but for whom there may still be fitness consequences from knock-on effects.
